# DNA methylation age acceleration and cognitive status among older adults in the US, 1999‐2002

**DOI:** 10.1002/alz70860_100927

**Published:** 2025-12-23

**Authors:** Erika Walker, Scarlet M Cockell, Belinda L Needham, Sung Kyun Park, Kelly M. Bakulski

**Affiliations:** ^1^ University of Michigan, Ann Arbor, MI, USA

## Abstract

**Background:**

Biological aging, measured by DNA methylation, is a potential biomarker for cognitive health outcomes. The purpose of this analysis was to evaluate associations between measures of aging using DNA methylation and cognition in a nationally representative sample of adults aged 60+ in the National Health and Nutrition Examination Survey (NHANES).

**Method:**

Annual cross‐sectional NHANES surveys (1999‐2002) with DNA methylation measures and cognitive testing were combined. Genome‐wide DNA methylation data were used to create 13 measures of biological aging trained on different aging phenotypes. Cognition was assessed with the Digit Symbol Substitution Test (DSST). Mild cognitive impairment was categorized as DSST score below the weighted 25th percentile. To evaluate the associations between each DNA methylation measure and continuous DSST score, survey weighted linear regression models adjusted for age, sex, race/ethnicity, education, smoking, serum cotinine, and BMI were run. Sensitivity analyses included assessing effect modification by sex, education, and race/ethnicity and running modified Poisson models with the binary mild cognitive impairment outcome. *p*‐values were adjusted using the false discovery rate method.

**Result:**

Included participants (*N* = 1,463) were an average of 70.5 years old, 82.7% non‐Hispanic White, and 71.6% high school educated or higher. The average DSST score was 46.9 points (maximum 117). 10 of 13 DNA methylation measures were associated with cognition measured by DSST (adjusted *p* <0.05). One year of GrimAge2 accelerated aging was associated with ‐0.41 points lower DSST score (95% CI: ‐0.59, ‐0.24; adjusted *p* = 0.001). One standard‐deviation decrease in estimated telomere length was associated with ‐2.19 points lower DSST score (95% CI: ‐1.16, ‐3.22; adjusted *p* = 0.001). In stratified analyses, higher magnitudes of association were observed among male and non‐Hispanic White participants across multiple aging measures. Mild cognitive impairment was observed for 39.7% of participants (DSST < 35). Associations between DNA methylation measures and mild cognitive impairment were not significant after multiple comparison adjustment.

**Conclusion:**

DNA methylation may be a useful biomarker of cognitive status among older adults.

